# Predicting the Benefits of Banana Bunchy Top Virus Exclusion from Commercial Plantations in Australia

**DOI:** 10.1371/journal.pone.0042391

**Published:** 2012-08-07

**Authors:** David C. Cook, Shuang Liu, Jacqueline Edwards, Oscar N. Villalta, Jean-Philippe Aurambout, Darren J. Kriticos, Andre Drenth, Paul J. De Barro

**Affiliations:** 1 Department of Agriculture and Food, Bunbury, Western Australia, Australia; 2 Cooperative Research Centre for National Plant Biosecurity, Bruce, Australian Capital Territory, Australia; 3 The University of Western Australia, Crawley, Western Australia, Australia; 4 Australian Centre for Biosecurity and Environmental Economics, Canberra, Australian Capital Territory, Australia; 5 CSIRO Ecosystem Sciences, Canberra, Australian Capital Territory, Australia; 6 Department of Primary Industries, Knoxfield, Victoria, Australia; 7 Department of Primary Industries, Parkville, Victoria, Australia; 8 Charles Sturt University, Barton, Australian Capital Territory, Australia; 9 The University of Minnesota, Minneapolis, Minnesota, United States of America; 10 The University of Queensland, Dutton Park, Queensland, Australia; 11 CSIRO Ecosystem Sciences, Dutton Park, Queensland, Australia; Virginia Tech, United States of America

## Abstract

Benefit cost analysis is a tried and tested analytical framework that can clearly communicate likely net changes in producer welfare from investment decisions to diverse stakeholder audiences. However, in a plant biosecurity context, it is often difficult to predict policy benefits over time due to complex biophysical interactions between invasive species, their hosts, and the environment. In this paper, we demonstrate how a break-even style benefit cost analysis remains highly relevant to biosecurity decision-makers using the example of banana bunchy top virus, a plant pathogen targeted for eradication from banana growing regions of Australia. We develop an analytical approach using a stratified diffusion spread model to simulate the likely benefits of exclusion of this virus from commercial banana plantations over time relative to a nil management scenario in which no surveillance or containment activities take place. Using Monte Carlo simulation to generate a range of possible future incursion scenarios, we predict the exclusion benefits of the disease will avoid Aus$15.9-27.0 million in annual losses for the banana industry. For these exclusion benefits to be reduced to zero would require a bunchy top re-establishment event in commercial banana plantations three years in every four. Sensitivity analysis indicates that exclusion benefits can be greatly enhanced through improvements in disease surveillance and incursion response.

## Introduction

Comprehensive bioeconomic decision support frameworks are increasingly needed to assist policy makers in managing plant biosecurity risks [1]. Benefit cost analysis is a highly effective means of communicating expected net returns from investment decisions to diverse groups of stakeholders [2]. For biosecurity economists, it can provide a valuable means to convey a raft of technical economic and scientific information via metrics that are easily understood by risk managers. In this paper, we demonstrate this important property using the example of Banana bunchy top virus (BBTV) in Australia, which is currently being considered for eradication from commercial growing areas.

Bananas are an important crop throughout the world, particularly in developing countries where their importance as a food crop is only surpassed by rice, wheat and maize [3]–[5]. More than 120 countries produce bananas, with world production estimated to be in excess of 100 million tonnes [4]. Australia contributes less than 0.5 per cent of global production [4], but banana cultivation makes a sizeable contribution to regional economies across northern Australia. In 2010, the States of Queensland, New South Wales, the Northern Territory and Western Australia produced a combined total of 301 450 tonnes of bananas with a gross value of Aus$492.2 million [6].

All commercially grown cultivars of banana have evolved as a result of intra-specific and inter-specific hybridsisation, parthenocarpy and triploidy, involving the two wild diploid species *Musa acuminata* and *Musa balbisiana* [5], [7]. Selection of high-yielding *Musa* clones and current agronomic practices in large-scale monoculture plantations has given rise to the occurrence of a wide range of pests and diseases [5], [8], of which BBTV is one of the most economically important. It causes stunted growth and infected plants rarely produce a bunch [9]. The virus is transmitted by the banana aphid (*Pentalonia nigronervosa*), as well as through infected plant suckers and other plant tissues used in banana propagation [10], [11].

BBTV has been present in eastern Australia since the early 1900s. Its severity was clearly demonstrated in the 1920s when approximately 90 per cent of the Queensland and New South Wales banana crops were destroyed [12]. This prompted State government initiatives to contain BBTV through eradication of infected plants and controls on the movement of planting material from affected areas, which led to a gradual recovery of the banana industry. In 1993, a five-year Banana Plant Health Improvement Project was initiated by the industry aimed at eradicating BBTV from Australia [13]. Despite achieving substantial reductions in the prevalence of the virus, outright eradication was not achieved by the end of this period.

In this paper, we examine a similar policy we term *exclusion*, which aims to remove the disease from banana producing regions and maintain their area freedom from the virus over time. We use computer-simulated economic impact scenarios to determine the likely net benefits of BBTV exclusion from commercial banana production areas. A stratified diffusion model is used to simulate BBTV prevalence and control responses under a nil management and a commercial exclusion scenario over time. We then compare these scenarios and calculate a likely financial return to the banana industry from adopting an exclusion strategy, and therefore the break-even level of investment industry and government can make in BBTV exclusion before the costs outweigh the benefits.

## Methods

We assume that the current presence of BBTV is eliminated from Australia commercial banana plantations and concentrate on events that might subsequently transpire. As such, we treat local eradication of future incursions in banana growing areas as an investment alternative to a nil management approach with respect to BBTV management. We assume that the Australian banana industry is represented by a single planning body determining appropriate biosecurity investment strategies. Predicted investment paths are defined as a function of expected yield and input cost changes (and hence profitability) from investing in BBTV exclusion relative to a nil management approach. We make the assumption that the planning body will choose to invest in BBTV exclusion in region (i.e. State or Territory) *i* in time step (i.e. year) *t* if it is expected to reduce grower losses by a greater amount than additional costs. The dichotomous adoption variable, 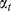, which takes on the value of 1 if the central planner invests in exclusion across *n* regions in year *t* and 0 otherwise, is defined as:


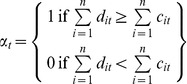
(1)

where 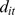 is the total difference in predicted cost increments induced by BBTV between the exclusion and nil management policy options in region *i* in time *t*, and 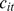 is the total cost of implementing an exclusion strategy in region *i* in time *t*. We focus on the estimation of 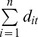 to determine how large 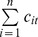 would need to be before 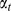 assumes a value of zero (i.e. the central planner does not adopt the exclusion policy). This approach is warranted given that the size of the investment to be made in BBTV exclusion has yet to be negotiated. It precents decision makers with a ceiling beyond which costs should not be considered lest a net loss in social welfare result.

The current pre-border biosecurity strategy for addressing the threat of exotic banana pathogens includes the use of strict phytosanitary measures on traded bananas, which lower the probability of BBTV re-entering an area via imported fruit. Indeed, these measures are so strict that they effectively mean prominent banana exporting countries such as the Philippines cannot land product in Australia at a sufficiently low price to be competitive on the domestic market for fresh bananas. Post-border biosecurity measures include monitoring through disease surveillance, detection and rapid response to incursions.

If, as a result of these post-border measures, a BBTV incursion in a commercial banana production area is detected early enough, there may be a strong likelihood of local eradication through plant removal and destruction. Hence, the value of 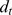 is influenced by local eradication costs and probability of eradication success. This probability of success is arbitrarily assumed to decline negative exponentially at an average rate of 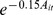, where 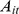 is the area infected with BBTV in region *i* year *t* weighted by the probability of infection and density of infection. We test the sensitivity of this assumption below using the range of values for the parameter of the negative exponential rate of decline of eradication success indicated in Table 1.

**Table 1:** Model parameters.

Description	Values
Probability of establishment, *z_aa_*. a	2.6×10^−4^ to 1.3×10^−1^
Detection probability.	Binomial(1.0, 0.6)
Exponential rate of decline for eradication success probability with respect to area affected	Pert(−0.20,−0.15,−0.10)
Population diffusion coefficient, *D* (m^2^/yr). a,b	Pert(0,2.5×10^3^, 5.0×10^3^)
Minimum area infected immediately upon entry, *A* ^min^ (m^2^).	1.0×10^3^
Maximum area infected, *A* ^max^ (m^2^). *^c^*	1.4×10^8^
Intrinsic rate of infection and density increase, *r*(yr^−1^). a	Pert(0.10,0.15,0.20)
Minimum infection density, *N* ^min^ (#/m^2^).	1.0×10^−4^
Maximum infection density, *K* (#/m^2^). a	Pert(100,550,1000)
Minimum number of satellite sites generated in a single time step, *S* ^min^ (#).	1.0
Maximum number of satellite sites generated in a single time step, *S* ^max^ (#). a	Pert(10,5,10)
Intrinsic rate of new foci generation per unit area of infection, *µ* (#/m^2^). a	Pert(1.0×10^−6^,3.0×10^−6^,5.0×10^−6^)
Discount rate (%).	5.0
Supply elasticity. *^d^*	Uniform(0.2,0.8)
Demand elasticity. *^d^*	Uniform(−1.1,−1.0)
Prevailing market price for bananas in the first time step ($/T). *^c^*	1,900
Maximum area considered for eradication (ha).	400
Cost of eradication, *E* ($/ha). *^e^*	Pert(1.0×10^4^,1.5×10^4^,2.0×10^4^)
Increased insecticide and application cost ($/ha). *^f^*	130
Yield reduction despite control, *Y* (%).	Pert(0.0,2.5,5.0)

a Specified with reference to Cook [30] and Waage et al. [36] using distributions defined in Biosecurity Australia [31]; *^b^* Derived from Sapoukhina et al. [37]; *^c^* ABS [6], Note 1ha  = 10 000 m^2^; *^d^* Ulubasoglu et al. [38]; *^e^* Assumes average density of planting of 2 000 stems/ha and removal, transport, destruction and chemical costs amounting to $20/tree. This is inclusive of labour (team of three at $50/hr per person), bulldozing equipment ($100/hr at 20 hr/ha), truck hire ($75/hr), incendiaries ($60/ha for green waste) and creation of a circular chemical buffer zone approximately 5 ha in diameter around previously infected sites. Chemical used is assumed to be dithane (applied at a rate of 3 kg/ha or $25/ha) and oil (applied at 3L/ha or $10/ha) at fortnightly intervals rotated with propiconazole (applied at a rate of 0.3L/ha or $5/ha). Assume 2 additional dithane treatments are required and 4 propiconazole treatments (and therefore 6 additional oil treatments), each taking 1 hr/ha to apply; *^f^* Assumes: (i) labour costs of $50/ha (i.e. 1 application × 1hr/ha × $50/hr); (ii) 75 mL of chemical solution is used per banana plant per treatment costing $10/L (e.g. dimethoate diluted to 75 mL/100L) (i.e. approximately $15/ha); and (iii) two additional chemical treatments will provide sufficient suppression of banana aphid [30].

If, under an exclusion policy, an outbreak occurs and is not detected early enough, local eradication within the area affected may be aborted. We assume that the decision on when to abort is based purely on affected area, and that a threshold exists beyond which local eradication is technically infeasible. When this threshold is reached, management effort switches to a longer term management strategy to slow the spread of BBTV using insect control technologies and lethal chemical treatments for infected plants. In such cases, the exclusion option fails.

Algebraically, we expressed 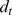 as:


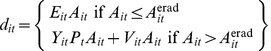


where: 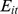 is the cost of eradication per hectare in region *i* in year *t*;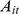, as stated above, is the area infected with BBTV in region *i* in year *t* weighted by the probability of infection and density of infection; 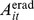 is the maximum technically feasible area of eradication in region *i* in year *t*; 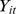 is the mean change in yield resulting from the control of insect vectors and treatment of infected plants in region *i* in year *t*; 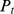 is the prevailing domestic price for bananas in year *t*; and 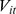 is the increase in variable cost of production per hectare induced by BBTV on-plantation management methods in region *i* in year *t*.


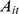
 is inclusive of BBTV re-entry and establishment probabilities, *z*, and therefore represents the area predicted to be in need of additional management effort (i.e. beyond normal plantation management activities) due to BBTV infection in region *i* in year *t*. The transition between a *with* disease (call it event *a*) and *without* disease (event *b*) state in the region is described as a regular Markov process such that the probability of event *a* occurring in any given time period will reduce to a constant value after several periods. Each element of the transition matrix 
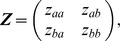
 where *a* defines the row and *b* the column, provides an indication of the susceptibility of the industry to the disease [14]. We use deterministic transitional probabilities, with 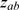 specified as the initial arrival probability, and 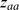
by an initial establishment probability. The remaining elements are 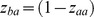 and 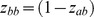.

If we denote the probabilities of the events *a* and *b* occurring at any time *t* by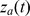 and 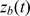, respectively, the probability of *a* occurring in time step 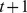 given that *b* has occurred in time step *t* can be expressed as:


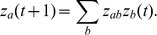


If 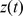
is a column vector with elements 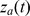
and 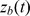, we can use the transition matrix to express equation (3) as:


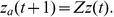


By applying this previous equation repeatedly, we obtain:


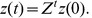


If our Markov chain is regular the vector 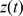
will converge to a unique vector 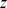 as *t* increases [15], [16]. Independent of the disease status of a region in time step *t*, we can accurately predict the probability of it being in either state *a* or *b* after several time periods, 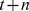. The probability of event *a* occurring in any given time period will reduce to a constant value after several time steps. Since we are only concerned with event *a* (i.e. BBTV occurrence) in a given region, *i*, we denote 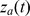 as 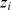.

To describe the movement of BBTV post-establishment in multiple regions we use a stratified diffusion model combining both short and long distance dispersal processes [17]. It is derived from the reaction diffusion models originally developed by Fisher [18] which have been shown to provide a reasonable approximation of the spread of a diverse range of organisms [19]–[23]. These models assert that an invasion diffusing from a point source will eventually reach a constant asymptotic radial spread rate of 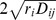 in all directions, where 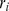 describes a growth factor for BBTV per year in region *i* (assumed constant over all infected sites) and 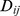 is a diffusion coefficient for an infected site *j* in region *i* (assumed constant over time) [17], [23]–[25]. Hence, we assume that the original infection (i.e. the first of a probable series of sites, *j*) takes place in a homogenous environment in region *i* and expands by a diffusive process such that area infected at time *t*, 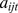, can be predicted by:


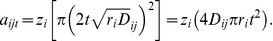


For practical purposes, an estimate of 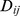 can be derived from the mean dispersal distance (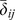) of the pathogen at an infection site, where 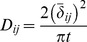 [26]–[28]. 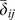 is the site-specific average distance (in metres) over which dispersal events leading to infection occur. By assuming 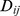 is constant across all sites *j* we ignore demographic stochasticity and consequent non-uniform invasion [27].

The density of BBTV infection within 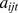 influences the control measures required to counter the effects of infection, and thus partially determines the value of 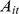. We assume that in each site *j* in region *i* affected, the infection density, 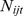, grows over time period *t* following a logistic growth curve until the carrying capacity of the environment, 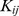, is reached:


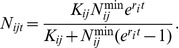


Here, 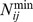 is the size of the original influx at site *j* in region *i* and 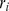 is the intrinsic rate of density increase in region *i* (assumed to be the same as the intrinsic rate of population increase) [27].

In addition to 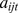 and 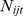, the size of 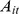 depends on the number of nascent foci (see Moody and Mack [29] – these are *satellite* infection sites) in year *t*, 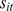, which can take on a maximum value of 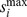 in any year. These sites result from events external to the outbreak itself, such as weather phenomena, animal or human behaviour, which periodically jump the expanding infection beyond the infection front [27]. We use a logistic equation to generate changes in 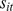 as an outbreak continues:


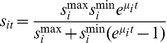


where 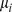 is the intrinsic rate of new foci generation in region *i* (assumed constant over time) and 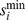 is the minimum number of satellite sites generated in region *i*.

Given the area of bananas affected by BBTV in different sites (i.e. given by equation (6)), the density of these infections (i.e. equation (7)) and the number of satellite sites they have created (i.e. equation (8)), we can calculate the total area (in hectares),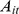, across *n* regions as:


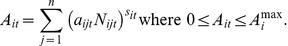


The total benefit to the central planner of adopting an exclusion policy for BBTV in year *t*, 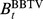, can be expressed as:


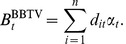


In the following section we estimate 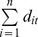 using multiple BBTV re-entry and spread scenarios for Australia’s banana growing regions over a 30 year period. These include grower areas of coastal Queensland, the north coast of New South Wales, parts of Western Australia and the Northern Territory (i.e. 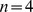) (see Table 2). Where there is uncertainty surrounding parameter values, they are specified within the model as distributions and a Latin hypercube sampling algorithm used to sample from each distribution. In each of 10 000 model iterations one value is sampled from the cumulative distribution function so that sampled parameter values are weighted according to their probability of occurrence. The model calculations are then performed using this set of parameters.

**Table 2:** Australian banana production statistics by region.

Producer	Area (ha)a,b	Production volume (MT) a,b	Average yield (T/ha)	Value produced (Aus$’000,000)^a^	Probability of entry, *z_ab_*
Queensland	12,234	338.6	27.7	448.3	Uniform(0.3,0.7)
New South Wales	1,372	13.9	10.2	17.7	Uniform(0.3,0.7)
Western Australia	200	5.6	28.2	15.1	Uniform(1.0×10^−6^, 1.0×10^−3^)
Northern Territory	203	6.0	29.5	11.1	Uniform(1.0×10^−6^, 1.0×10^−3^)

a ABS [6].

b Australian Banana Growers’ Council.

Table 2 provides banana production information for each region used in the analysis. It also contains region-specific BBTV (re-)entry and (re-)establishment probabilities. Given the continued stringent SPS measures against imported bananas, the probability of entry into new areas beyond the historical distribution of BBTV (i.e. Northern Territory and Western Australia) is regarded as very low: within the range 1.0×10^−3^ to 5.0×10^−2^ [30]. In areas where the virus has been present (i.e. Queensland and New South Wales), the likelihood of re-entry was arbitrarily assumed to be low: within the range of 5.0×10^−2^ to 0.3 [31]. The probability of establishment upon entry was assumed to be moderate in all regions: within the range of 0.3 to 0.7 [30].

A list of all other model parameter distributions appears in Table 1. Note that *i*, *j* and *t* subscripts are omitted in Tables 1 and 2 since, with the exception of *z_ab_*, *z_aa_* and insecticide and application cost, parameter specification does not change over spatial or temporal ranges. Table notes provide details where a spatial variation is assumed.

## Results

**Figure 1:**
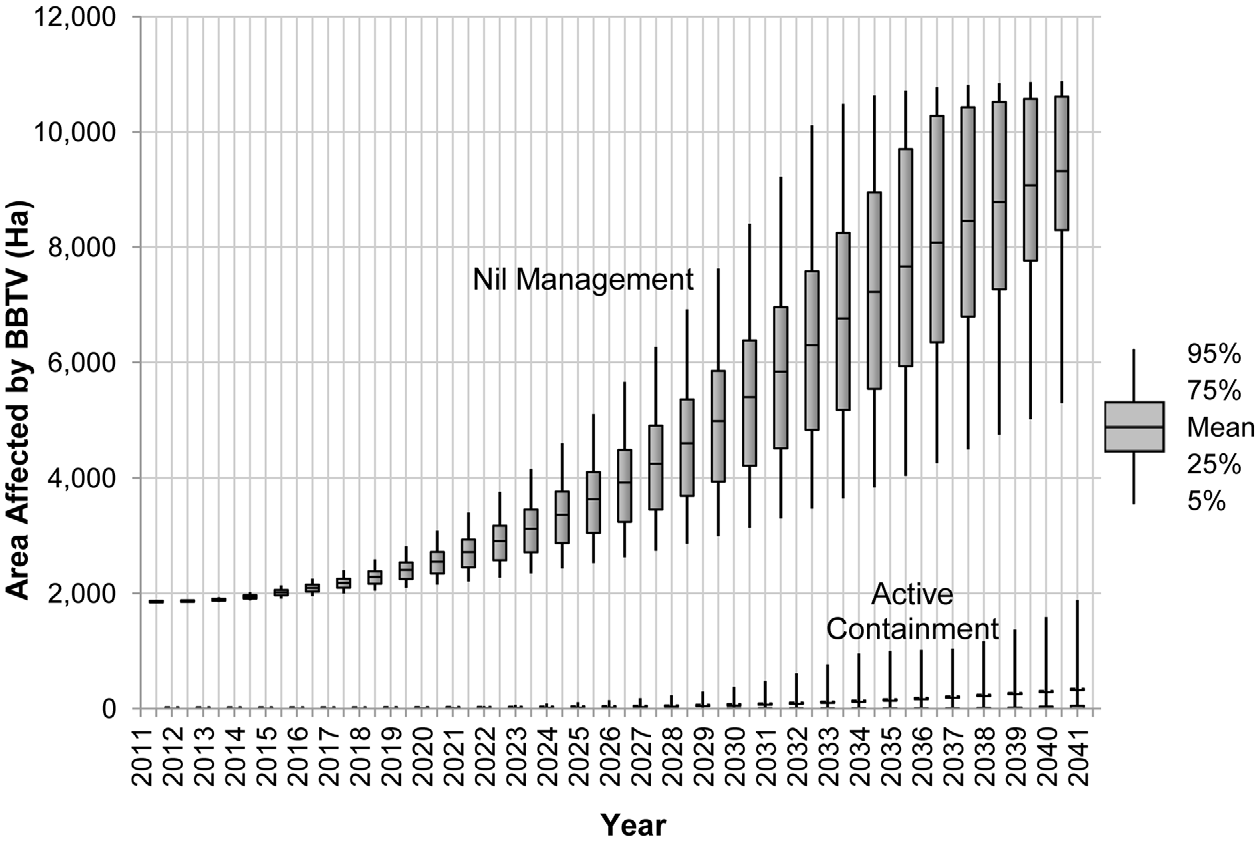
Likely spread of BBTV over time with and without an active containment policy.

Despite exclusion from commercial production areas being assumed to have been achieved at the outset of the analysis, our assumptions are such that re-establishment is likely to occur at some point or multiple points over the estimation period. The model simulates these re-establishment events as a Poisson process where BBTV successfully re-establishes in Queensland and New South Wales on an average of one year in six, and in Western Australia and the Northern Territory one year in 50. Therefore, the resultant expected spread area values under the exclusion and nil management scenarios calculated from the 10 000 iterations of the model are positive. However, as Figure 1 reveals, the extent of expected spread under an exclusion or active containment program is substantially below that of a nil management policy due largely to the local eradication measures in place immediately upon detection. These projections have been aggregated across all production regions to produce Figure 1.

The present value of benefits accruing from the exclusion of BBTV from commercial plantations is estimated by the model to average Aus$18.9 million per year over 20 years across banana producing regions (i.e.
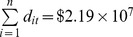
). This represents the threshold level of 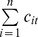 beyond which the central planning body will choose not to invest in an exclusion strategy as an alternative to a nil management strategy (i.e. 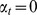). The standard deviation of the distribution of average annual biosecurity benefits is Aus$3.5 million and skewness −1.4 (i.e. the distribution is skewed left such that the left tail is long compared to the right tail). Given current average banana yields, our estimated value of 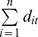 is equivalent to an annual avoidance of losses in national banana production harvest volume of 12 thousand tonnes per year.

**Figure 2:**
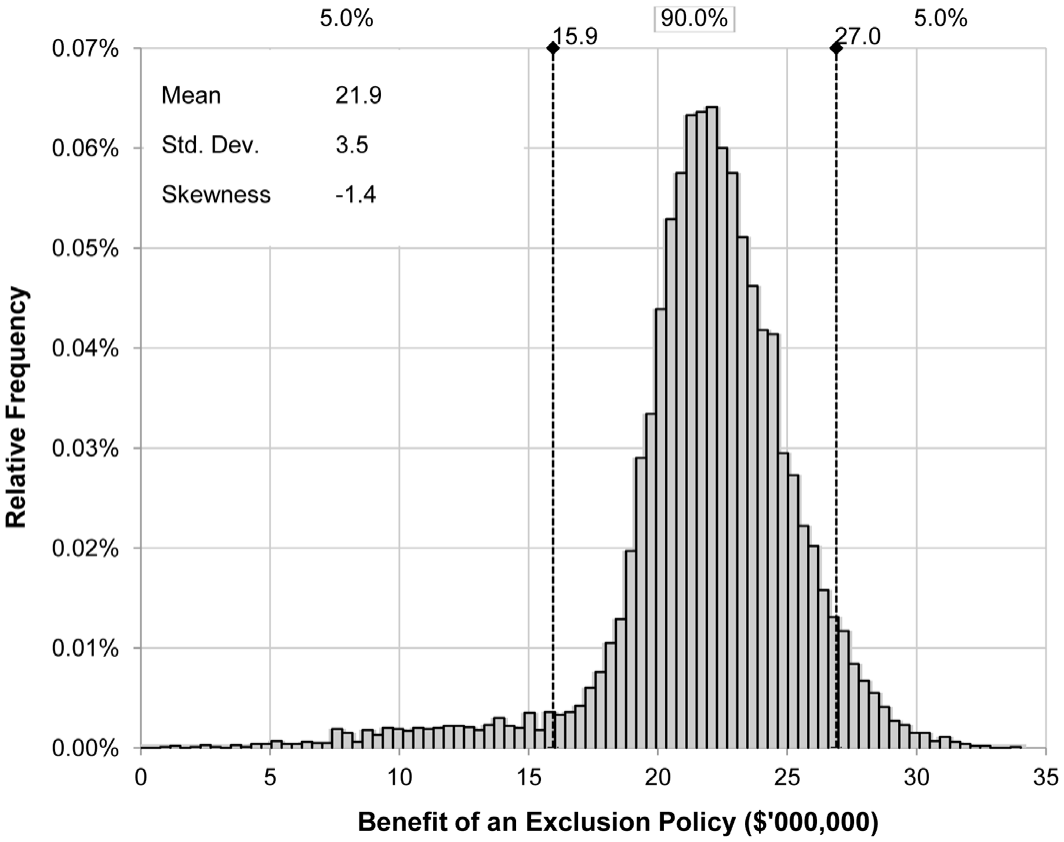
Expected annual benefit of a BBTV exclusion policy.

**Figure 3:**
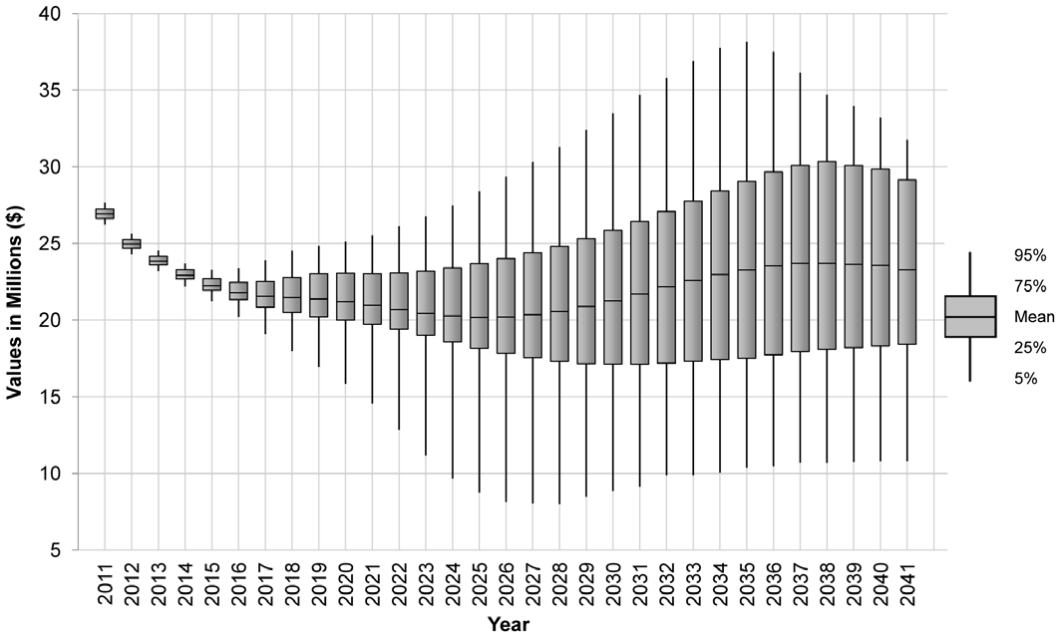
Expected annual benefit of BBTV exclusion over time.

While Figure 2 shows average benefits over a 20-year period, Figure 3 illustrates how these annual exclusion benefits are expected to change over a 30-year period. Here, the mean benefit of BBTV exclusion predicted by the model is plotted with confidence bounds. All projected benefits are discounted at 5 per cent per annum [32]. Initially, the benefits of exclusion fall due to the erosive effects of the discount rate. But, they begin to rise as the expected difference in BBTV prevalence between the exclusion and nil management scenarios increases the further into the future we project.

**Figure 4:**
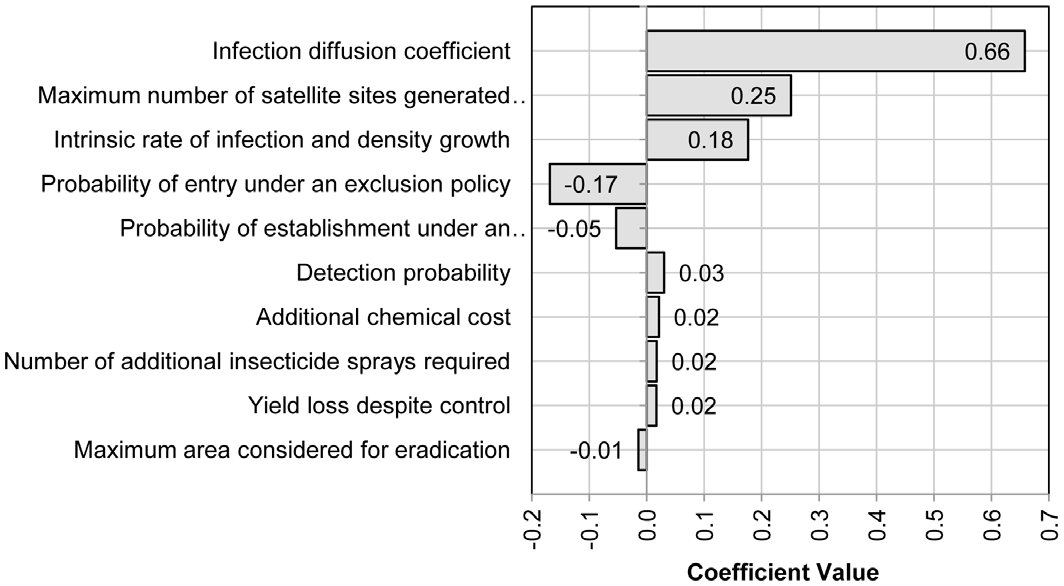
Sensitivity analysis.

In view of the uncertainty surrounding many of the parameters used to describe the BBTV (re)infection and spread process, we use multivariate stepwise regression to test the statistical significance of each input variable on 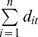. The input variables shown in Figure 4 are listed in descending order of statistical significance, and their corresponding regression coefficients indicate the effect a one unit increase in each will produce in terms of units of 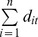 (i.e. dollars per year) if all other inputs are held constant. The R-squared value for this analysis, 0.60, indicates that inputs in the model explain 60 per cent of the variance in 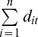.

The sensitivity test indicates that the model is responsive to changes in ten of the parameters listed in Tables 1 and 2. The most significant input variable is the infection diffusion coefficient which has a regression coefficient of (0.66). This implies that if all other variable are held constant, each 1.0 m^2^/yr increase in the mean of the infection diffusion coefficient (i.e. 2 500 m^2^/yr in our model) increases the mean of 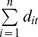 by $0.66. Other sensitive parameters and their corresponding regression coefficients include the maximum number of satellite sites generated in a single time step (0.25), the intrinsic rate of infection and density growth (0.18), the probability of entry under an exclusion policy (−0.17), the probability of establishment under an exclusion policy (−0.05) and detection probability (0.03).

## Discussion

Our results are indicative of the potentially large benefits of investing in active containment of BBTV. Based on the model outlined in the Methods section, it is shown in the Results section that excluding the virus from commercial production areas is likely to produce a net benefit over time provided the annual costs of doing so do not exceed Aus$21.9 million.

The most sensitive model parameter (i.e. the infection diffusion coefficient) cannot be influenced by policy. It is a parameter determined by biological characteristics of the pathogen and those of its principal host. Hence, its high level of sensitivity indicated by our model is of little relevance to policy makers. But, other sensitive parameters can be influenced by policy at different phases of BBTV re-infection. In Table 3, we have separated those that can be influenced by policy from those that cannot, and of those that can be influenced by policy decisions we indicate which phase of re-infection will influence parameter values. For convenience, their regression coefficients are also repeated in brackets.

**Table 3:** Policy and non-policy input parameters.

Phase of BBTV re-infection	Policy	Non-policy
Pre-border/border	Probability of entry under an exclusion policy (−0.17)	
Post-border	Maximum number of satellite sites generated in a single time step (0.25)Probability of establishment under an exclusion policy (−0.05)Detection probability (0.03)Additional chemical cost (0.02)Maximum area considered for eradication (−0.01)	Infection diffusion coefficient (0.66)Intrinsic rate of infection and density growth (0.18)Number of additional insecticide sprays required (0.02)Yield loss despite control (0.02)

Unlike the infection diffusion coefficient, the maximum number of satellite sites generated in a single time step can be influenced by post-border biosecurity policies that reduce incidences of the virus being transferred from infected to uninfected plantations. This can be achieved through targeted extension that informs growers and industry employees of the risk of moving plants and equipment between growing areas without appropriate disinfection procedures. Hence, this input variable is grouped as a post-border policy variable in Table 3.

From Figure 4, we can deduce that the probabilities of entry and (to a lesser extent) establishment under an exclusion policy are relatively important in determining the total benefit of an exclusion policy. Table 3 indicates that the former can be influenced by pre-border phytosanitary measures, although the main source of re-infection may be domestic sources rather than foreign. The probability of establishment under an exclusion policy partially depends on the effectiveness of post-border biosecurity measures that influence the probability that BBTV comes into contact with viable hosts.

However, both the probabilities of entry and establishment would need to be high before the benefits of exclusion are reduced to zero. In other words, industry and government would need to do an extremely poor job of maintaining area freedom from the virus once it has been removed from commercial plantations for the exclusion approach to fail entirely. To indicate how high the probability of BBTV entry and establishment under an exclusion strategy must be to produce a result where the central planner is indifferent between the exclusion and nil management options (i.e._
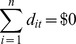
_) requires the model to be aggregated across all States and Territories. If we consider the sum of all banana growing areas in Australia as one susceptible host block, the probability of BBTV entry and establishment under an exclusion strategy that would lead to expected costs in both policy scenarios to be equivalent is approximately 0.75. This requires a re-entry and establishment event to occur in a commercial plantation in three of every four years.

The sensitivity of total exclusion benefits to the detection probability is lower than that of the probability of entry under an exclusion policy, indicating that in the case of BBTV prevention may be better than cure. So, in the absence of cost information, Table 3 suggests that once the virus is removed from commercial plantations investment in its on-going exclusion may be more effective in maintaining area freedom than periodic “stamp out” policies to remove infections when they occur.

But, improving the ability of plantation owners, quarantine inspectors and plant pathologists to detect the virus may be a cheaper strategy than attempting to lower the re-entry probability. Given Australia’s history with BBTV and the visibility of disease symptoms, raising the detection probability may only require increases in the frequency or intensity of surveillance rather. It follows that strategies that encourage plantation monitoring and disclosure of detection information could increase the likely returns of an active containment strategy over time.

Whether the costs of such strategies would outweigh the benefits requires a separate analysis focused specifically on surveillance. Given the sensitivity of some of the non-policy parameters listed in Table 3, particularly the infection diffusion coefficient and the intrinsic rate of infection and density growth, further investigation is also required to reveal the likely costs of influencing these parameters.

As mentioned in the introduction to the paper, the cost of achieving complete BBTV exclusion from commercial banana growing regions is not known, but we can speculate what they might be with the aid of a historical example. The eradication of the fungal pathogen black Sigatoka (*Mycosphaerella fijiensis* (Morelet)) from north Queensland was achieved between 2001 and 2003. *M. fijiensis* was detected in 2001 in the Tully area, the major banana-growing region of Australia. Although previous detections of the fungus in far north Queensland were eradicated with similar tactics to those we have suggested for local BBTV eradication (i.e. destruction of infected plants), a program of intensive de-leafing was employed to remove the majority of inoculum from plants in the Tully outbreak [33], [34]. This was followed by intensive fungicide treatment applied to plants weekly in rotation for a period of 6 months after de-leafing. In total, the eradication cost was Aus$17 million [34].

If this figure can be considered broadly representative of a relatively small scale eradication program, let us hypothetically assume that the exclusion of BBTV might involve a cost more than three times this amount. Even if exclusion costs from commercial production areas are as high as Aus$60 million and it takes a full five years to remove the virus completely, our results indicate that returns to the industry would be highly favourable. A benefit cost analysis performed using our estimated value would produce a benefit cost ratio of 1.8∶1.0 (i.e. every $1.00 spent on eradicating the disease returns $1.80 worth of benefit to the industry). It is possible, indeed likely, that exclusion of BBTV from the main production areas can be achieved at substantially lower cost. If this is the case and exclusion is achieved, the returns on investment will be significantly higher.

Future extension of the model developed in this analysis could include the consideration of feedback effects of BBTV to the regional and national economies using a general equilibrium model [35]. While the importance of potential costs of non-market (e.g. environmental costs due to the use of pesticides) and indirect market impacts (e.g. reduced purchases of inputs after an industry is affected) of BBTV are acknowledged, they have not been included in the model. If the environmental costs of the use of, for instance, pesticides to control BBTV insect vectors were to be included, the benefits of exclusion over time would probably increase. Using a general equilibrium model or using an ecosystem services approach may improve the investigative power of the analysis, but would impose a cost in terms of the increased need for information to run the models.
